# Advanced Materials in 3D/4D Printing Technology

**DOI:** 10.3390/polym14163255

**Published:** 2022-08-10

**Authors:** Houwen Matthew Pan

**Affiliations:** Division of Chemistry and Biological Chemistry, School of Physical and Mathematical Sciences, Nanyang Technological University, 21 Nanyang Link, Singapore 637371, Singapore; matthew.pan@u.nus.edu

Advances made in 3D printing have opened new avenues for innovation in dental, aerospace, soft robotics, thermal regulation, and flexible electronic devices. Current state-of-the-art for 3D printing technologies include fused deposition modelling (FDM), selective laser sintering (SLS), direct ink writing (DIW), and stereolithography (SLA). Recently, 4D printing was borne from combining 3D printing technology with stimulus-responsive printing ink. Four-dimensional-printed structures evolve as a function of time and exhibit intelligent behavior such as self-assembly and actuation. To match the rapid development of 3D and 4D printing technologies, a myriad of printing materials with advanced functionalities have also been invented.

In this Special Issue, original research articles focusing on the study and creation of advanced materials for 3D/4D printing technologies have been collected. Dynamic, bio-inspired spider silks were successfully fabricated via the 4D printing of shape memory polyurethane. The shape morphing behaviors of bio-inspired spider silks were programmable via pre-stress assemblies enabled by 4D printing. Copper complexes were synthesized as novel visible light photoinitiators for the free radical photopolymerization of acrylates at the safe wavelengths of 405 and 455 nm. The new photoinitiators were used for direct laser write experiments. A facile, water-based 3D-printable ink with sustainable nanofillers and cellulose nanocrystals (CNCs) was developed for DIW to 3D print strong PI/CNC composite aerogels for advanced thermal regulation. Dental forceps were 3D printed via fused filament fabrication (FFF) and continuous fiber reinforcement (CFR) technologies with comparable properties to conventional devices in terms of extraction force. Three layers of corrugated PVC gel artificial muscles were 3D printed using a multi-material, integrated direct writing method and displayed good actuating performance with potential applications in soft robotics and flexible electronic devices. Using FDM technology, moulds were prepared for the casting of carbon fiber components. By combining FDM with chemical smoothing, low-cost moulds with a high quality surface finish were successfully produced. Micro-/nanostructures were 3D printed on surfaces with different wetting properties, and biomolecules with different functionalities were integrated into the base polymer ink. The ability to incorporate binding tags to achieve specific interactions between relevant proteins and the fabricated micro-/nanostructures, without compromising mechanical properties, paves the way for numerous biological and sensing applications.

Additionally, in-depth research of 3D printed components for specific applications has also been included. Studies were carried out on the sound reflection behavior of four different types of 3D-printed, open-porous polylactic acid (PLA) material structures, which found that sound reflection behavior was strongly affected by the type of material structure, excitation frequency, the total volume porosity, the specimen thickness, and the air gap size. Polyphenylsulfone polymer powders were evaluated as fire-resistant materials for processing by SLS. The microstructural evaluations and the mechanical property results indicate sufficient intra- and inter-layer consolidation together with reasonable tensile property responses. Lastly, a homogeneous structural element was generated that accurately represents the behavior of FDM-processed materials, using a representative volume element (RVE). The homogenization summarizes the main mechanical characteristics of the actual 3D printed structure, enabling the accurate engineering analysis of the final 3D-printed structure.

Finally, the Editors express their appreciation to all contributors to this Special Issue and hope that it inspires and guides scientists to achieve greater progress in the area of Advanced Materials for 3D and 4D Printing.

